# Efficacy of Low-Dose Rituximab on Neuromyelitis Optica-Associated Optic Neuritis

**DOI:** 10.3389/fneur.2021.637932

**Published:** 2021-05-04

**Authors:** Shuo Zhao, Huanfen Zhou, Quangang Xu, Hong Dai, Shihui Wei

**Affiliations:** ^1^Department of Ophthalmology, Beijing Hospital, National Center of Gerontology, Institute of Geriatric Medicine, Chinese Academy of Medical Sciences, Beijing, China; ^2^Department of Neuro-Ophthalmology, The Chinese People's Liberation Army General Hospital, Beijing, China

**Keywords:** neuromyelitis optica, optic neuritis, rituximab, immunosuppression, aquaporin 4 antibody

## Abstract

**Purpose:** To prospectively investigate the efficacy and tolerance of low-dose rituximab (RTX) for the treatment of neuromyelitis optica-associated optic neuritis (NMO-ON).

**Methods:** Optic Neuritis patients with seropositive aquaporin 4-antibody (AQP4-Ab) were diagnosed with NMO-ON and recruited for treatment with low-dose RTX (100 mg ^*^ 4 infusions) and were then followed monthly for a minimum of 3 months. Reinfusion of 100 mg RTX was given when the CD19+ B lymphocyte frequency was elevated to above 1%. The serum AQP4-Ab level was tested by an enzyme-linked immunosorbent assay (ELISA).

**Results:** A total of 43 NMO-ON patients (1 male/42 female, 75 involved eyes) were included in this study. CD19+ B cell clearance in the peripheral blood was induced in 97.7% of patients after induction treatment. A significant decrease in serum AQP4-Ab concentration was observed after induction treatment (*P* = 0.0123). The maintenance time of B cell clearance was 5.2 ± 2.25 months. The relapse-free rate was 92.3% in patients followed-up for over 12 months, and patients with non-organ-specific autoimmune antibodies tended to relapse within 6 months. A total of 96.2% of patients had stable or improved vision, and a decrease in the average expanded disability status scale (EDSS) score was found. Structural alterations revealed by optic coherence tomography were observed in both ON and unaffected eyes. The rates of infusion-related reactions and long-term adverse events (AEs) were 18.6 and 23.1%, respectively. No severe AEs was observed.

**Conclusions:** Low-dose rituximab is efficient and well-tolerated in treating NMO-ON.

## Introduction

Neuromyelitis optica-associated optic neuritis (NMO-ON) is an inflammatory autoimmune optic neuropathy with severe visual loss. Relapse may occur in 90% of NMO-ON patients, which may lead to poor visual outcome and can be accompanied by progressing neurological disability (1, 2). Therefore, the most critical management strategy in the remission phase of NMO-ON is to control clinical relapses, thereby improving the prognosis.

Neuromyelitis optica-associated optic neuritis is diagnosed with a positive serum aquaporin-4 antibody (AQP4-Ab), which targets the astrocytic water channel in the central nervous system (CNS) (3). Aquaporin-4 antibody-mediated autoimmunity is considered to play the most critical role in the pathogenesis of NMO (4, 5). In the last decade, the monoclonal antibody rituximab (RTX) targeting B cells has been gradually applied for the management of relapses in Neuromyelitis optica spectrum disorders (NMOSDs) (5–7). Previous studies have suggested that RTX could significantly reduce the annual relapse rate (ARR) in approximately 90% of patients, improve the degree of disability, shorten the length of spinal cord lesions, and show good safety (6–10). Intravenous RTX, as an empirical immunotherapy in treating NMOSDs, has been listed as the first-line treatment in the remission phase (11, 12).

Regimens of RTX treatments in NMO were based on the use of RTX by patients with lymphoma (13) (375 mg/m^2^ infused once per week for 4 weeks or 1,000 mg infused twice, with a 2-week interval), which were high-cost, off-label therapies, and might have a high risk of adverse reactions. In recent years, several studies have indicated that a reduced dose of RTX has therapeutic value for NMOSD, mostly based on the evaluation of expanded disability status scale (EDSS) scores and ARR (9, 14, 15). Expanded disability status scale scores, known as a classic disability assessment method in NMOSD, were insufficient for patients with ON as the main attack. The decrease in monocular vision might not even change the EDSS score. Furthermore, few studies have paid attention to the fluctuation of serum AQP4-Ab levels during the treatment procedure after low-dose RTX treatment. The relationship of AQP4-Ab levels with disease activity after low-dose RTX treatment remains unclear.

This study is the first to administer low-dose RTX to NMO-ON patients in a Chinese neuro-ophthalmologic center. In this study, comprehensive visual function evaluation was performed, risk factors associated with disease activity were evaluated. Neuromyelitis optica-associated optic neuritis was diagnosed with seropositive AQP4-Ab using cell-based assays (CBAs), and the fluctuation of antibody levels was tested using an enzyme-linked immunosorbent assay (ELISA) method throughout the follow-up period.

## Materials and Methods

### Patients

Hospitalized patients diagnosed with relapsing NMO-ON in the remission phase were enrolled in the Chinese People's Liberation Army General Hospital (PLAGH) from February 2017 to August 2019 for this study. Neuromyelitis optica-associated optic neuritis was diagnosed with seropositive AQP4-Ab using both CBAs and ELISA methods (16). Patients with hepatic or retinal diseases, cardiac dysfunction, a history of cancer and chronic infection, abnormal blood cell count, pregnancy, and refractive error exceeding −6.0 D were excluded. The patients were followed for at least 3 months. Failure to follow up for 2 consecutive months was considered follow-up loss. All adverse events (AEs) during RTX treatment were recorded.

### Treatment Protocol

In the induction phase, 100 mg of RTX was intravenously (IV) administered once a week 4 consecutive times. Peripheral blood B-cell counts (using CD19 expression) were obtained at baseline and every month. Further infusions were given at 100 mg IV when the B cell frequency was elevated to above 1%. All patients were pretreated with ibuprofen 0.3 g orally and promethazine hydrochloride 25 mg IV prior to each RTX infusion. High-dose methylprednisolone IV (IVMP) (1g/day for 3–5 days) was used for the management of acute disease attack.

### Tests for Serum AQP4-Ab and Other Autoimmune Antibodies

For qualitative test of AQP4-Ab, we established a transfected cell line of EGFP-AQP4-m23-HEK293 using the published methods (16). The patient's serum was gradiently diluted and add to the fixed cells. Positive results were determined when fluorescent labeling of anti-human second antibody coincided with EGFP. For all NMO-ON patients, an ELISA method (RSR Co., US) was used to test AQP4-Ab at baseline and after RTX treatment at least once every 2 months. A flow cytometry method was used to detect the frequency of CD19+ B cells in peripheral blood before and after the first infusion, after the induction treatment (four infusions) and then per month. Blood was also drawn for total antinuclear antibody (ANA) titers and autoantibodies against double-stranded DNA, extractable nuclear antigens including Sjögren syndrome A (SSA)/B (SSB), ribosomal p protein, Scl-70, Jo-1, thyroglobulin (TG), thyroid peroxidase (TPO), and β2-glycoprotein I antigen in the rheumatologic research center in PLAGH.

### Ophthalmological Examinations, OCT Procedures, and Evaluation of EDSS Score

Ophthalmologic examinations included slit lamp inspection, swinging-light tests for the search for RAPD, and direct or indirect ophthalmoscopy with dilated pupils for retinal examination. Best-corrected visual acuity (BCVA) was tested using a Snellen chart.

OCT was performed using Cirrus HD-OCT (software version 3.0, Model 4000; Carl Zeiss Meditec, Inc., Dublin, CA, USA). All OCT scanning was performed by an experienced operator in a darkroom. Patients with a pupil diameter <2 mm received mydriasis. We followed OSCAR-IB criteria in the retinal OCT quality assessment (17). Peripaillary retinal nerve fiber layer (pRNFL) thickness was calculated by averaging the following four quadrants: temporal, nasal, superior, and inferior. Macular data were evaluated by the cube average thickness (quadrant measurements of retinal thickness in a 6 × 6 mm volume cube between the inner limiting membrane and the retinal pigment epithelium: ILM-RPE) and the thickness map displaying measurements calculated from nine macular areas corresponding to the Early Treatment Diabetic Retinopathy Study (**Figure 5**).

Expanded disability status scale scores were evaluated in all NMO-ON patients using the Kurtzke Functional System Rating Scale. Two doctors evaluated the same patient separately and calculated the average EDSS score at each evaluation.

### Statistical Analysis

Statistical analyses were performed using the Statistical Program for Social Sciences statistical software (version 21.0; IBMSPSS, Inc., Chicago, IL). The numeric variable is represented as Mean ± SD. A paired *t*-test was used to compare measurement data in the follow-up period with those at baseline. The differences before and after treatment in the two groups were compared by analysis of variance (ANOVA) or rank-sum test. Categorical data were analyzed using the chi-squared test or Fisher's exact test. *P*-values < 0.05 were considered significant.

## Results

### Demographic Manifestations

A total of 43 patients with NMO-ON (75 eyes) were included in this study. The average age at the time of enrollment was 33.5 ± 12.79 years, and females accounted for 97.7% (42/43). At the time of enrollment, 23 patients only experienced ON. The last attack was ON in 36 patients and or ON combined with acute myelitis in seven patients. The time from the last attack to enrollment was 2.4 ± 1.63 months. Ten patients were treated with immunotherapies before enrollment, including seven of azathioprine (oral 1.5–4.0 mg/Kg^*^days), one of mycophenolate mofetil (MMF, oral 1.5–3.0 g/days), and two of MMF combined low-dose prednisolone. A total of 12 patients had accompanying autoimmune diseases (ADs) at the time of enrollment (12/43, 27.9%), including seven organ-specific (OS) ADs (autoimmune thyroid disease, idiopathic thrombocytopenic purpura, myasthenia gravis) and five non-OS (NOS) ADs (systemic lupus erythematosus and Sjögren syndrome). Twenty-two patients (22/43, 48.8%) had other autoimmune antibodies. The frequency of combined OS autoantibodies (OS-Abs: TG-Ab, TPO-Ab) was 25.6% and that of combined NOS autoantibodies (NOS-Abs: ANA, SSA, etc.) was 37.2%. The demographic and clinical details of NMO-ON are summarized in Table 1.

**Table 1:** Demographic and clinical manifestations of NMO-ON patients.

Numbers of patients, *n*	43
Age at enrollment, years, mean ± SD (range)	33.5 ± 12.79(13–55)
Age at onset, years, mean ± SD (range)	28.5 ± 11.81(10–55)
Sex ratio, M:F	1:42
Ethnicity	All Han Chinese
Involved eyes, *n*	75
Clinical characters of ON	
One episode, *n* (%)	6(14.0)
Multiple episodes, *n* (%)	37(86.0)
Unilateral involved, *n* (%)	11(25.6)
Bilateral involved, *n* (%)	32(74.4)
First episode	
ON, *n* (%)	37(86.0)
Myelitis, *n* (%)	4(9.3)
Other core clinical symptoms, *n* (%)	2(4.7)
Disease duration, months, mean ± SD (range)	58.2 ± 62.79(3–270)
Average EDSS score, mean ± SD	2.2 ± 1.12
Immunosuppression treatments before enrollment^a^, *n* (%)	
None or oral low-dose prednisolone	33(76.7)
AZA	7(16.3)
MMF	1(2.3)
MMF combined prednisolone	2(4.7)
Accompanied autoimmune diseases, *n* (%)	12(27.9)
HT	6(14.0)
SS	4(9.3)
SLE	1(2.3)
ITP	1(2.3)
MG	1(2.3)
Accompanied autoimmune antibodies, *n* (%)	22(48.8)
ANA	13(30.2)
TG-Ab	9(20.9)
TPO-Ab	9(20.9)
SSA/SSB-Ab	12(27.9)
a-β2-GPI-Ab	3(7.0)
Anti-ribosomal p protein Ab	1(2.3)

a *Treatment for at least 3 months*.

*NMO-ON, neuromyelitis optica associated optic neuritis; RTX, rituximab; AZA, azathioprine; MMF, mycophenolate Mofetil; HT, Hashimoto's thyroiditis; SS, Sjögren syndrome; SLE, systemic lupus erythematosus; ITP, idiopathic thrombocytopenic purpura; MG, myasthenia gravis; ANA, antinuclear antibody; TG-Ab, anti-thyroglobulin antibody; TPO-Ab, anti-thyroid peroxidase antibody; SSA/SSB-Ab, Sjögren syndrome A (SSA)/B (SSB) antibody; a-β2-GPI-Ab, anti-β2-glycoprotein I antibody*.

### Relapses and ARR

**Figure 1:**
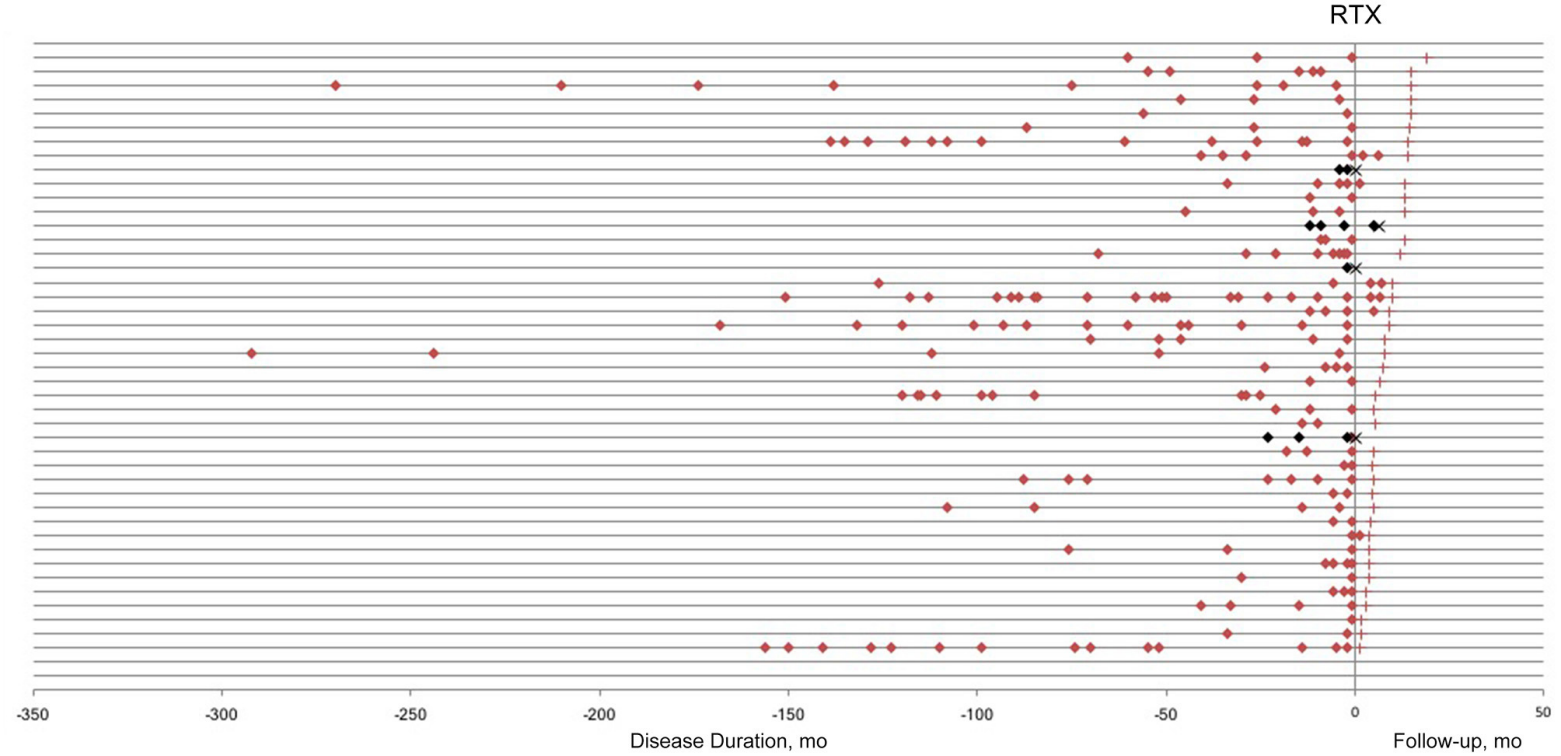
Relapses in neuromyelitis optica-associated optic neuritis patients before and after low-dose rituximab (RTX) treatment. Each horizontal line represents a patient. Red/Black square, relapse; red cross, last follow-up; black cross, lost to follow-up.

The patients were followed up for 3–19 months (median 8 months). A total of 11 relapses in eight patients, including two ON and nine acute myelitis, were observed during the follow-up period. The average time from induction to first recurrence was 3.3 ± 2.32 months (ranged 0.25–6 months). The relapses before and after low-dose RTX treatment are presented in Figure 1. Among 13 patients who were followed up for more than 1 year, the ARR decreased significantly from 1.19 ± 0.286 to 0.15 ± 0.154 (*p* = 0.009), and the relapse-free rate was 92.3% (12/13).

A total of 22 patients were followed up for more than 6 months. Of the 22 patients, six relapsed. The comparison of clinical characteristics between relapsed and non-relapsed patients indicated a higher frequency of NOS-Abs in relapsed patients (*p* = 0.046) (Table 2).

**Table 2:** Comparison of clinical characters between relapsed and non-relapsed patients within 6 months of RTX treatment.

	**Relapsed (*N* = 6)**	**Non-relapsed (*N* = 16)**	***p***
Serum AQP4-Ab concentration deduction percentage after RTX Induction, %	30.6	40.6	0.624
Age at Onset, years, mean ± SEM	27.0 ± 3.04	24.6 ± 2.52	0.598
ARR before enrollment^a^, mean ± SEM	1.58 ± 0.428	0.96 ± 0.158	0.101
Combined with autoimmune diseases, *n* (%)	3(50.0)	3(18.8)	0.283
Combined with autoimmune antibodies, *n* (%)	5(83.3)	8(50.0)	0.333
NOS-Abs, *n* (%)	5(83.3)	4(25.0)	0.046^*^
OS-Abs, *n* (%)	0(0.0)	5(31.3)	–

a *Calculated based on data of 21 patients whose disease course was more than 1 year*.

* *P < 0.05 (Fisher exact probability test)*.

*RTX, rituximab; AQP4-Ab, aquaporin-4 antibody; NOS-Abs, non-organ specific antibodies; OS-Abs, organ specific antibodies*.

### Dynamic Changes of B Cells and Serum AQP4-Ab

**Figure 2:**
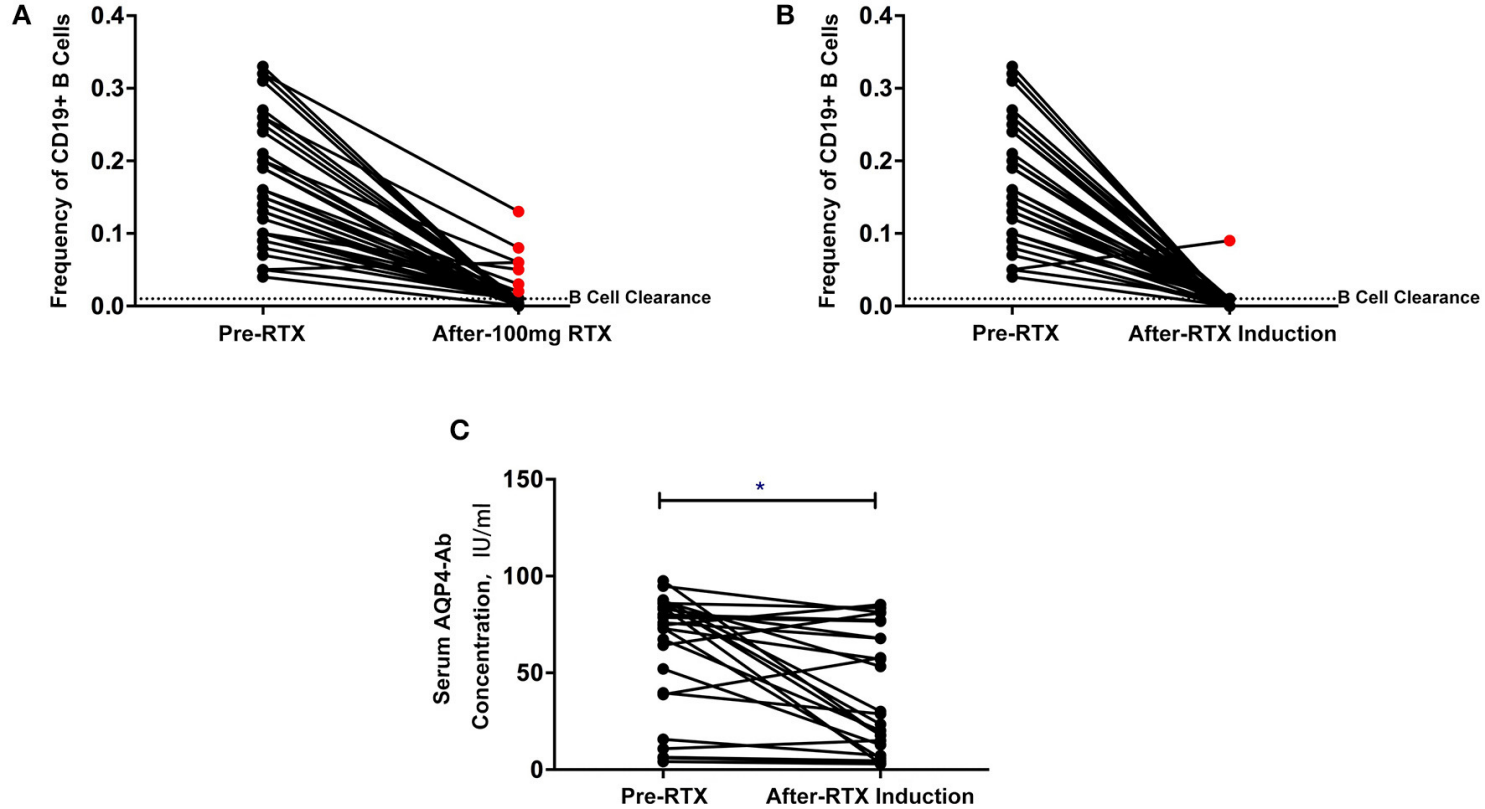
**(A,B)** Changes in CD19+ B cell frequency before and after the first rituximab infusion **(A)** and induction treatment **(B)** (*red point*: patients without B cell clearance). **(C)** Changes in serum aquaporin-4 antibody (AQP4-Ab) before and after low-dose rituximab induction treatment. *P < 0.05.

Complete B-cell clearance (CD19+ B cell ≤1%) was observed in 35 patients (81.4%) after the first RTX infusion and 42 patients (97.7%) after the induction treatment (Figures 2A,B). One patient experienced an increase in the frequency of B cells after the first infusion and relapsed within a short period of time after induction treatment (**Figure 4B**).

The maintenance time of B cell clearance ranged from 2 to 12 months (directly into the second cycle of treatment) within 1 year after induction (5.2 ± 2.25 months). Reinfusion was administered in 22 patients, of whom 20 were followed up for 6 months or more. The average treatment interval was 4.4 ± 2.26 months. Most of the reinfusion occurred in the eighth month after induction treatment (46.2%) (Table 3).

**Table 3:** Presentation of the re-infusion time after RTX induction treatment.

**Time after RTX induction treatment, months**	**1**	**2**	**3**	**4**	**5**	**6**	**7**	**8**	**9**	**10**	**11**	**12**
*N*^a^	29	23	25	15	13	12	11	13	7	11	4	13
Patients of Re-infusion, *n*	0	1	5	6	3	5	3	6	1	4	1	5
Re-infusion percentage, %	0.0	4.3	20.0	**40.0**	23.1	**41.7**	27.3	**46.2**	14.3	36.4	25.0	38.5

a *Patients undergone test for Serum AQP4-Ab concentration*.

*The re-infusion percentage over 40% was presented in bold font*.

**Figure 3:**
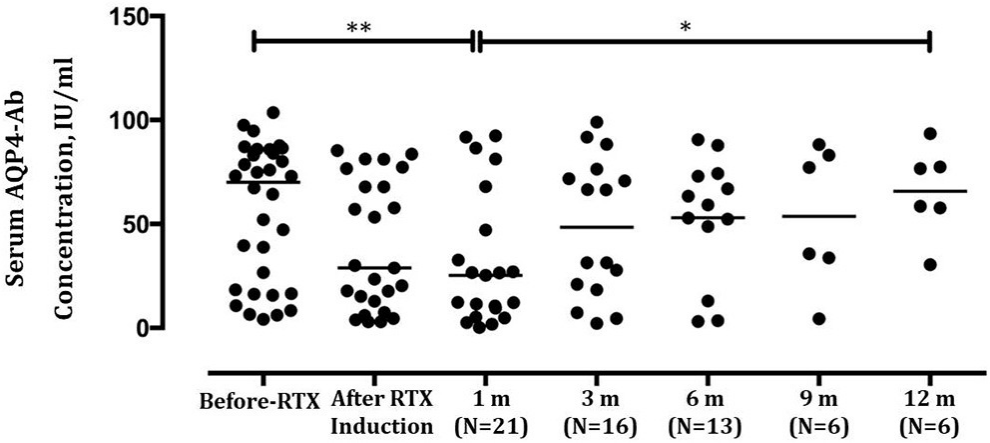
Comparison of serum aquaporin-4 antibody (AQP4-Ab) levels in neuromyelitis optica-associated optic neuritis patients before and after low-dose rituximab induction within 12 months. **P < 0.01; *P < 0.05.

The overall serum AQP4-Ab levels decreased significantly after induction treatment (*P* = 0.0123), but AQP4-Ab level in four patients elevated (Figure 2C). The fluctuation of serum AQP4-Ab levels in 12 months is shown in Figure 3. Compared with baseline, the serum AQP4-Ab level decreased significantly after 1 month (*P* = 0.009) but increased significantly after 12 months of induction treatment (*P* = 0.025).

**Figure 4:**
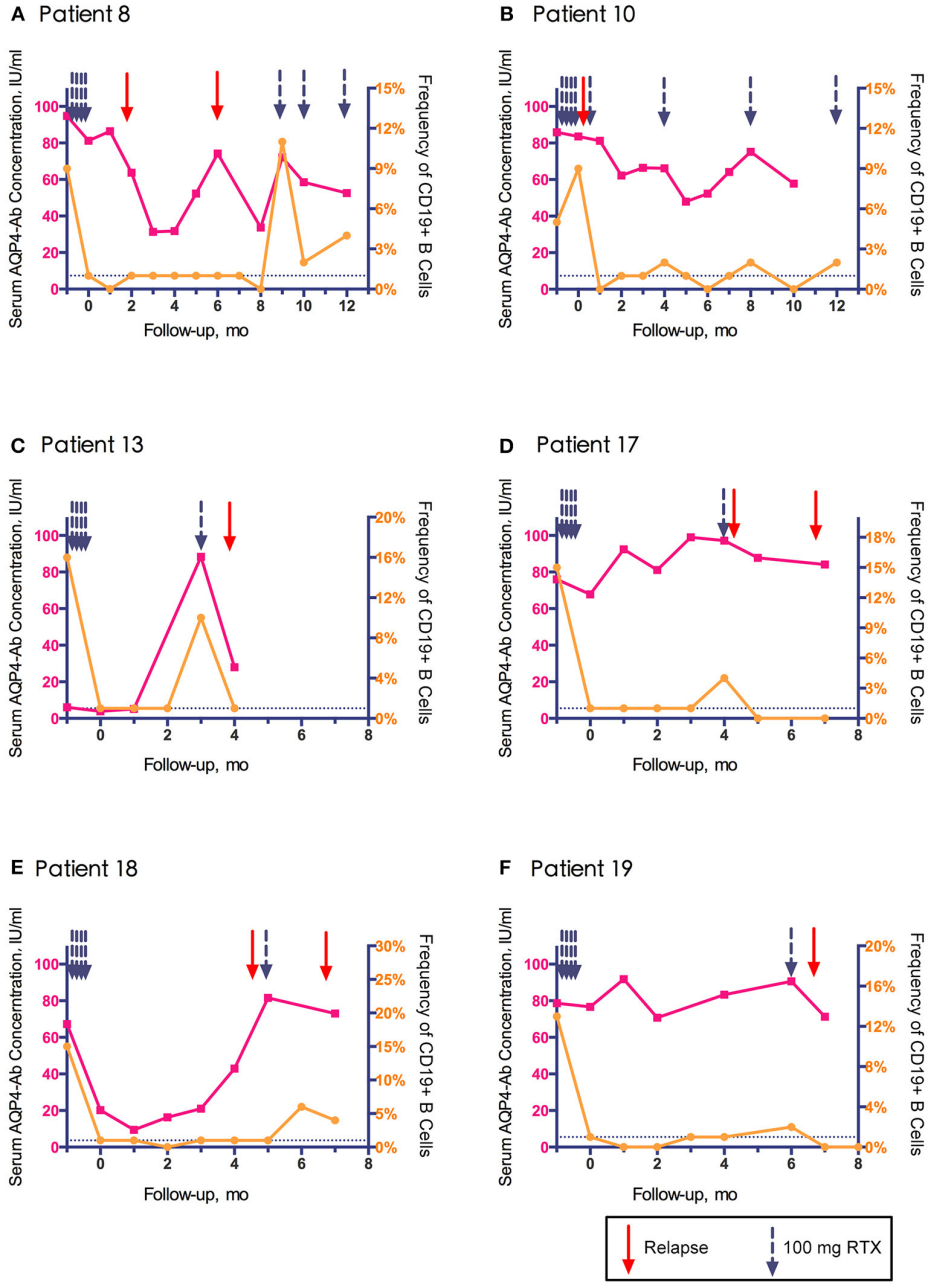
Association of clinical relapse with CD19+ B cell frequency and aquaporin-4 antibody (AQP4-Ab) level in six relapsed patients with neuromyelitis optica-associated optic neuritis.

Among the 11 relapses, 6 (54.5%) were accompanied by B cell regeneration (ratio>1%), and 5 (45.4%) occurred within 14 days after RTX infusion. AQP4-Ab was tested in 10 relapses, of which 9 (90%) showed rapidly increased or continuous high levels of AQP4-Ab. The peripheral blood CD19+ B cell frequency and serum AQP4-Ab level in relapsed patients are shown in Figure 4 (data from patient No. 24 are not shown because AQP4-Ab was not detected at relapse). The increase in AQP4-Ab could occur regardless of the regeneration of CD19+ B cells.

### Ophthalmological Findings and EDSS Scores

A total of 13 patients (26 eyes) were followed up for at least 1 year, of whom BCVA, OCT parameters and EDSS scores after 1 year of treatment were compared with those at enrollment. The results showed that BCVA improved in six eyes (23.1%), remained stable in 19 eyes (73.1%), and was reduced in only one eye (3.8%). The average EDSS score of the 13 patients was 2.85 ± 0.291 after treatment for 1 year compared with 3.00 ± 0.291 at baseline (*p* = 0.219).

**Figure 5:**
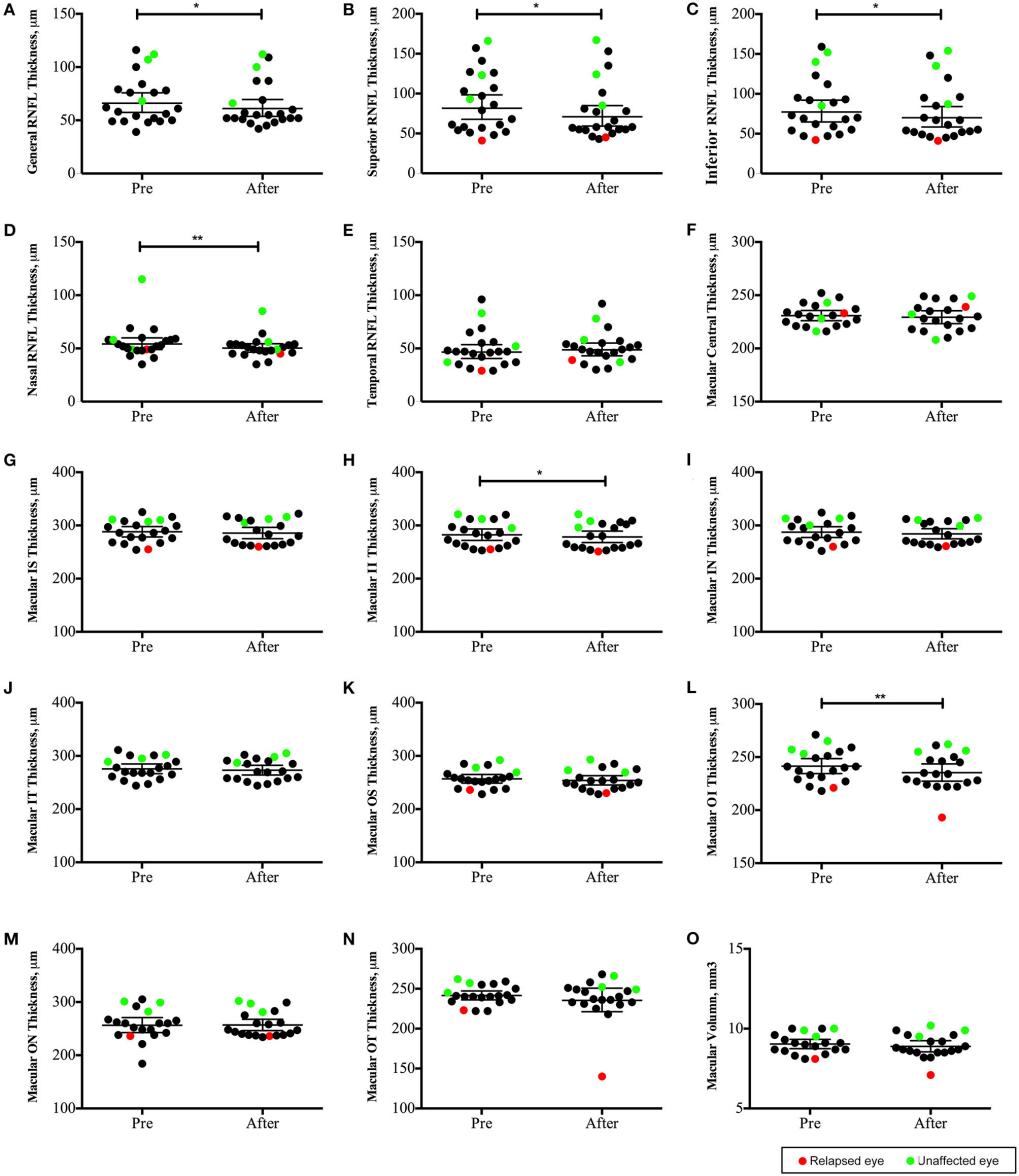
Changes in optic coherence tomography (OCT) parameters in neuromyelitis optica-associated optic neuritis patients followed up for 12 months (22 eyes): The peripapillary retinal nerve fiber layer (pRNFL) was significantly thinner in the superior, inferior and nasal quadrants **(B–D)**; the macular neuroretinal thickness was significantly decreased in the inferior quadrant of both the inner and outer rings **(H,L)**. **P < 0.01; *P < 0.05. Red points: relapsed eyes; Green point: unaffected eye. IS, superior quadrant of the inner ring; II, inferior quadrant of the inner ring; IN, nasal quadrant of the inner ring; IT, temporal quadrant of the inner ring; OS, superior quadrant of the outer ring; OI, inferior quadrant of the outer ring; ON, nasal quadrant of the outer ring; OT, temporal quadrant of the outer ring).

OCT data was collected in 22 eyes (invalid data of four eyes with low vision was excluded). The peripapillary retinal nerve fiber layer (pRNFL) was significantly thinner in the superior, inferior, and nasal quadrants (Figures 5B–D); the macular neuroretinal thickness was significantly decreased in the inferior quadrant of both the inner and outer rings (Figures 5H,L). Moreover, structural alterations were observed in unaffected eyes (Figure 5, green points).

### AEs and Severe AEs

There were a total of 8 (18.6%) mild infusion reactions reported in patients, including chills, nasal congestion, sore throat, fatigue, elevated body temperature, and dizziness. Seven cases occurred at the first infusion, and one case occurred at the fifth infusion. Spontaneous remission was noted in all of cases. Two cases of pulmonary infection and one case of urinary infection were observed in the 13 patients followed up for over 1 year, and all recovered after antibiotic treatment. There were no cases of SAEs observed throughout the observation period.

## Discussion

In this study, low-dose RTX was prospectively applied to NMO-ON patients for the first time. According to the 2015 diagnostic criteria of NMOSDs (18), 43 AQP4-Ab seropositive NMO-ON patients were included in this study. At present, the detection methods of AQP4-Ab include CBA, tissue section immunofluorescence staining, flow cytometry, ELISA, and radioimmunoassay. Cell-based assay is considered to have higher sensitivity and specificity compared to ELISA and other method (16). So we used CBA for qualitative test of AQP4-Ab. In this study, all patients tested positive for AQP4-Ab by both CBA and ELISA methods, which ensured the accuracy of diagnosis and the consistency of our cohort. The average age of onset in this group was 28.5 years old, which was younger than that reported in other studies, and the majority (97.7%) were female. This might be due to the bias caused by the younger patients, who were more likely to accept new treatment methods, which could not represent the demographic characteristics of NMOSD. However, high prevalence rates of multiple episodes and bilateral involved ON were observed in our cohort, which was in accordance with previous studies of NMO-ON (2).

Here, we found that after 1 year of low-dose RTX treatment (approximately 20% of the conventional dose), the ARR decreased significantly, and the relapse-free rate was 92.3%. Up to 96.2% of patients had stable or improved vision, and a decrease in the average EDSS score was found. The prognostic visual acuity in NMO-ON was found to be correlated with the time of acute treatment at ON onset, as well as ON relapses (19). So the decreased ARR could contribute to the favorable prognosis. In a study of conventional-dose RTX for NMOSD, the reduction rate of ARR ranged from 25% to 100% (6–8, 10, 20). We considered that the studies with relatively low effectiveness of RTX have the following commonalities: (1) patients included in most of the studies were diagnosed with NMO or long segment myelitis, which had higher EDSS scores than our ON patients; (2) positive serum AQP4-Ab was not included in the inclusion criteria in many studies, and patients with negative AQP4-Ab may have different pathological mechanisms, such as myelin oligodendrocyte glycoprotein antibody or glial fibrillary acidic protein antibody mediated autoimmunity (21–23); and (3) in some studies, RTX was used in refractory NMO treatment. Patients uncontrolled by other immunosuppression therapies might have higher disease activity. In our study, all patients were AQP4-Ab seropositive, and 76.7% of the patients had not used immunosuppressive therapy in the past. Therefore, this study might provide stronger clinical evidence of the usage of low-dose RTX for NMOSD treatment.

The dynamic changes in B cells and the fluctuation in serum AQP4-Ab were the focus of this study. We observed a high percentage of B cell clearance after either 100 mg RTX alone or induction treatment. The average time for B cell regeneration, which was not presented in small-sample studies of similar RTX dosages (9, 14), was 5.2 months. Greenberg et al. compared B cell regeneration time between different doses of RTX for B cell clearance (<2% of CD19+ B cells) and found that the maintenance time (average 6.1 months) after a single administration of 1,000 mg was significantly longer than 100 mg (average 3.3 months) (24). As the induction RTX dose was 400 mg in our study, the maintenance time of B cell clearance was between the two dosages. The proportion of reinfusion within 6 months after induction (90.9%) in our study is similar to that in other conventional dose studies. The results in our study indicated the similar efficacy of low-dose RTX to higher-dose RTX treatment on B cell clearance. In addition, low-dose RTX treatment was better than conventional dose treatment with respect to health care costs. In our cohort, the maximum dose of RTX applied for 12 months of follow-up was only 700 mg, which was less than half of the conventional dose.

However, while demonstrating the effectiveness of RTX, we found individual differences among patients with RTX treatment (Figure 4), which was consistent with conventional dose treatment (25, 26). Of the 11 episodes of relapse, five occurred within 2 weeks of the last RTX administration; the underlying baseline disease activity might contribute to the early relapses. Besides, application of RTX was considered to stimulate disease activity. Pellkofer et al. reported a synergistic increase in B cell-stimulating factor and AQP4-Ab in serum within a few days after RTX application (27), and another study speculated that monocytes were activated and proinflammatory cytokines were released after immature B cells were cleared (28). Therefore, there is a possibility that the application of RTX will cause short-term disease activation in some patients. In other studies, the heterogeneity of NMOSD patients' response to RTX treatment could also be observed, along with the worsening of the disease after RTX infusion (29–31). Kim et al. observed 100 NMO patients after RTX treatment and found that patients with different responses to treatment had FCGR3A gene polymorphisms. The FCGR3A-158F gene sequence may be related to incomplete clearance and short-term regeneration of memory B cells (32). Li et al. considered that the appearance of anti-RTX antibodies may lead to resistance to RTX treatment (15). However, the relationship between B cell regeneration and disease recurrence is still unclear. In our study, 45.4% of clinical recurrences in this study occurred with peripheral blood CD19+ B cell clearance. Previous studies have suggested that the activation of NMO pathogenic B cells occurs before CD19+ B cells, and it might be more accurate to monitor CD27+/CD20+ or class-switched memory B cells in subsequent studies (8, 33, 34).

Additionally, this study presented the fluctuation of serum AQP4-Ab concentration in NMO-ON and analyzed the correlation between relapse and AQP4-Ab. We found that 90% of clinical recurrences were accompanied by persistently high levels of serum AQP4-Ab or rapid elevation, which provided evidence of the relationship between AQP4-Ab and disease activity. Kim et al. presented the association of relapses with AQP4-Ab levels in nine NMO patients and concluded the temporal association of clinical relapses with increases in AQP4-Ab levels. However, 1/3 of the patients included in their studies were seronegative for AQP4-Ab, which might lead to bias in the analysis (26). This study also found that NMO-ON could be stable with high levels of AQP4-Ab, indicating that other related factors, such as the integrity of the blood-brain barrier, complement, and other cytokines, may also contribute to the pathological process.

The risk factors for patients with relapse within 6 months were analyzed in this study, and we found that the frequency of NOS-Abs in relapsed patients (83.3%) was higher than that in non-relapsed patients (25.0%; *P* = 0.044). The relationship between NOS-Abs and NMO recurrence is controversial. It was reported that NOS-Abs were unrelated to ARR and the severity of NMO (35), but it was also found that ANA-positive NMOSD patients have a reduced recurrence frequency and a better prognosis (36). However, this study is the first to report the relationship between NOS-Abs and NMO recurrence after RTX treatment, and a larger sample size study is needed to verify the results.

This study also dynamically observed the changes in OCT parameters in patients within 1 year. Significantly decreased thicknesses in the superior, inferior, and nasal quadrants of the pRNFL and in the inferior quadrant of macular were observed. Although OCT has been used for the evaluation of ON in NMO or MS in many studies, longitudinal observation studies have been rare. It is reported that NMO-ON eyes have lower peri-papillary retinal nerve fiber layer and macular ganglion cell + inner plexiform layer thicknesses, as well as a “flater” disc when compared with MS-ON eyes (21, 37). Moreover, while MS-ON mostly affected the small-diameter neurons in the temporal quadrant of the optic disc, NMO-ON more affected the nerve fibers above and below the optic disc (38, 39), consistent with the results in this study. In addition, we also observed that the patient's “unaffected eye” may also have gradual loss of retinal nerve fibers, indicating the subclinical involvement of the “unaffected” optic nerve in NMO-ON (40).

The proportion of AE in this study was lower than that in most conventional-dose studies (6, 8, 26, 27). In addition, no SAEs leading to discontinuation of treatment were observed in this study. Therefore, low-dose RTX treatment was generally well-tolerated in NMO-ON patients.

In summary, we reported the efficacy of low-dose RTX on the recurrence frequency of NMO-ON. The recurrence of NMO-ON was related to the ratio of CD19+ B cells in peripheral blood and the continuous high- or short-term increase in serum AQP4-Ab, and patients with NOS-Abs might tend to relapse early (within 6 months) after RTX induction. Low-dose RTX was well-tolerated in our cohort with a low proportion of AEs. Limitations existed in this study due to the uncontrolled design and relatively short follow-up time. Moreover, we were unable to draw a survival curve for possible risk factors for relapses due to the short follow-up time of 6 months. A multicenter randomized controlled trial comparing different RTX treatment strategies with other immunosuppression treatments for NMO-ON is needed in the future.

## Data Availability Statement

The raw data supporting the conclusions of this article will be made available by the authors, without undue reservation.

## Ethics Statement

The studies involving human participants were reviewed and approved by The Chinese People's Liberation Army Hospital Ethics Committee. Written informed consent to participate in this study was provided by the participants' legal guardian/next of kin.

## Author Contributions

QX and SZ contributed to the study design. HZ and SZ contributed to the data collection. HD contributed to the data analysis and interpretation. SW contributed to the manuscript preparation. SZ provided fundings to this research. All authors contributed to the article and approved the submitted version.

## Conflict of Interest

The authors declare that the research was conducted in the absence of any commercial or financial relationships that could be construed as a potential conflict of interest.
